# ‘ARMAN’ archaea depend on association with euryarchaeal host in culture and in situ

**DOI:** 10.1038/s41467-017-00104-7

**Published:** 2017-07-05

**Authors:** Olga V. Golyshina, Stepan V. Toshchakov, Kira S. Makarova, Sergey N. Gavrilov, Aleksei A. Korzhenkov, Violetta La Cono, Erika Arcadi, Taras Y. Nechitaylo, Manuel Ferrer, Ilya V. Kublanov, Yuri I. Wolf, Michail M. Yakimov, Peter N. Golyshin

**Affiliations:** 1 School of Biological Sciences, Bangor University, Deiniol Road, Bangor, LL57 2UW UK; 2 Immanuel Kant Baltic Federal University, Kaliningrad, 236040 Russia; 3 National Center for Biotechnology Information, National Library of Medicine—National Institutes of Health, Bethesda, MD 20894 USA; 4 Winogradsky Institute of Microbiology, Research Center for Biotechnology Russian Academy of Sciences, Prospect 60-Letiya Oktyabrya 7/2, Moscow, 117312 Russia; 5 Institute for Coastal Marine Environment, CNR, Spianata S. Raineri 86, 98122 Messina, Italy; 6 Insect Symbiosis Research Group, Max Planck Institute for Chemical Ecology, Hans-Knöll-Strasse 8, Jena, 07745 Germany; 7 Institute of Catalysis CSIC, Campus Cantoblanco, 28049 Madrid, Spain

## Abstract

Intriguing, yet uncultured ‘ARMAN’-like archaea are metabolically dependent on other members of the microbial community. It remains uncertain though which hosts they rely upon, and, because of the lack of complete genomes, to what extent. Here, we report the co-culturing of ARMAN-2-related organism, Mia14, with *Cuniculiplasma divulgatum* PM4 during the isolation of this strain from acidic streamer in Parys Mountain (Isle of Anglesey, UK). Mia14 is highly enriched in the binary culture (ca. 10% genomic reads) and its ungapped 0.95 Mbp genome points at severe voids in central metabolic pathways, indicating dependence on the host, *C. divulgatum* PM4. Analysis of *C. divulgatum* isolates from different sites and shotgun sequence data of Parys Mountain samples suggests an extensive genetic exchange between Mia14 and hosts in situ. Within the subset of organisms with high-quality genomic assemblies representing the ‘DPANN’ superphylum, the Mia14 lineage has had the largest gene flux, with dozens of genes gained that are implicated in the host interaction.

## Abstract (web summary)

In the absence of complete genomes, the metabolic capabilities of uncultured ARMAN-like archaea have been uncertain. Here, Golyshina et al. apply an enrichment culture technique and find that the ungapped genome of the ARMAN-like archaeon Mia14 has lost key metabolic pathways, suggesting dependence on the host archaeon *Cuniculiplasma divulgatum*.

## Introduction

Deep metagenomic analysis of environmental samples from acidic environments across our planet has demonstrated the existence of previously neglected uncultured archaea that are only very distantly related to recognised phyla^1^. Initially detected at Iron Mountain (California, USA), these archaeal lineages were subsequently confirmed to occur in various acid mine drainage (AMD) systems^2^. This enigmatic group of archaea (the so-called ‘Archaeal Richmond Mine Acidophilic Nano-organisms’, or ‘ARMAN’ was initially found in the fraction of cells filtered through 0.22 μm membrane filters^1^. Metagenomic assemblies suggested average genome sizes of these organisms to be relatively small for free-living organisms (approximately 1 Mbp)^1^. An interesting observation documented by electron microscopy was that some cells of a small size (<500 nm) interact through pili-like structures with larger cells that lacked cell walls. Comolli and colleagues^3^ suggested the ‘ARMAN’ organisms were the ‘small’ cells, whereas cell wall-deficient larger cells were attributed to some members of the order Thermoplasmatales, a group of organisms known to be widely represented in AMD systems^4^. Emerging findings from metagenomic data sets of ARMAN-like archaea and especially their ubiquity suggest that this group plays important roles in the environment, although the exact roles have yet to be established^2^.The phylogenomic placement of archaea from this group still represents a matter for discussion^5–7^.

The known example of small-sized cultured archaea is represented by *Nanoarchaeum equitans*, currently the only validly described member of the phylum Nanoarchaeota. Cells are about 500 nm (or smaller) in diameter and exhibit atypical archaeal ultrastructure^8^. *Nanoarchaeum equitans* exists only in association with the host, *Ignicoccus hospitalis*, which supplies certain organic compounds (lipids and amino acids), growth factors and likely ATP to *N. equitans* ^9^. Other nanoarchaeota-related examples include an Nst1 archaeon forming an association with its host, the *Sulfolobales-*related organism^10^, and ‘*Candidatus* Nanopusillus acidilobi’, thriving in a partnership with *Acidilobus* spp.^11^. These nanoarchaeota are hyperthermophilic marine and terrestrial organisms with extremely compact genomes that likely are not of an ancestral nature, but rather probably resulted from massive gene loss^6^. Nanoarchaeota-related organisms (including those known only by metagenomics-resolved genomes) are phylogenetically clustered within the ‘DPANN’ candidate superphylum (abbreviated after candidate divisions ‘Diaphetotrites’, ‘Parvarchaeota’*, ‘Aenigmarchaeota’, ‘Nanohalarchaeota’* and the only validly described phylum Nanoarchaeota)^12^. Recently, a number of uncultured ‘DPANN’ archaea with almost complete genomes were predicted by Castelle and co-authors^13^ to be symbiotic and/orto have a lifestyle based on fermentation. To summarise, all experimentally validated examples of interactions between co-cultured small (or ‘nanosized’) archaea and their partners are limited to Crenarchaea being the hosts. All of them (except *Ignicoccus* sp.) are acidophiles, while so far no associations have been co-cultured or characterised for Euryarchaeota, except those from the recent report on a four-member consortium containing a fungus, two strains of Thermoplasmatales and ARMAN-1-related organism with, due to the complexity of this enrichment culture, only a partially sequenced genome^14^.

Here, we report the co-cultivation and analysis of the ungapped genome of an ARMAN-like organism, the ‘*Candidatus* Mancarchaeum acidiphilum’ Mia14, which was enriched in the laboratory binary culture with *Cuniculiplasma divulgatum* PM4, a recently described representative of the family Cuniculiplasmataceae within Thermoplasmata^15^. After additional sampling campaigns and de novo metagenome sequencing of the microbial community of the acidic streamer of Mynydd Parys/Parys Mountain, we revealed possible in situ interactions of these organisms with other microbial community members. Furthermore, we analysed the voids in its metabolic pathways (and thus dependencies on potential hosts) and mapped its phylogenetic position. Finally, using data on arCOGs gains and losses, we reconstructed its evolutionary trajectory starting from the last archaeal common ancestor (LACA), which pointed at Mia14 having the greatest known extent of gene fluxes within the ‘DPANN’ superphylum.

## Results

### Coexistence of Mia14 with *Cuniculiplasma divulgatum* PM4

We have previously isolated and described two strains of a new archaeal family, genus and species, named *Cuniculiplasma divulgatum* (order Thermoplasmatales), from acidic streamers at Parys Mountain (UK) and Cantareras mine (Spain)^15^. Both strains S5 and PM4 were characterised as acidophilic organoheterotrophes with mesophilic optima for growth and as facultativeanaerobes^15^. The genomes of these isolates were remarkably similar to one another (>98% average nucleotide identity^16^ and to that of the genomic assembly ‘G-plasma’ from Iron Mountain (USA))^17^. During the isolation, *C. divulgatum* strain PM4 was co-cultured for 2 years with another archaeon designated Mia14 with a proportion of genomic reads, PM4:Mia14 of approx. 10:1. The initially poor growth of the PM4 component was significantly improved by the addition of complex organic compounds, such as beef extract and trypton (0.1% w/vol). However, the enhanced growth of *C. divulgatum* and an increased frequency of re-inoculations had a dramatic effect on the growth of Mia14, which was eliminated from the culture and, after approximately 2.5 years of regular (every 20–22 days) passages into the fresh medium, was not detectable by PCR with specific primers. Another possible explanation is that the faster growth of *C. divulgatum* strain PM4 was the result of the elimination of Mia14, which may have negatively affected the growth of PM4 in earlier cultivation stages. Whatever the case, we could not maintain Mia14 for longer than 2.5 years. However, as Mia14 was highly enriched in the initial enrichment cultures with *C. divulgatum* strain PM4, we obtained enough coverage of its genomic reads (approximately 40-fold) to assemble a single chromosome. After the loss of Mia14 from the enrichment culture, we performed additional sampling of the acidic streamer (from the same site in Parys Mountain where the isolate PM4 was derived from), and detected Mia14 initially by PCR using specific primers, then by the de novo sequencing of environmental DNA, and ultimately, by catalysed reporter deposition fluorescence in si﻿tu hybridisation (CARD-FISH).

**Fig. 1:**
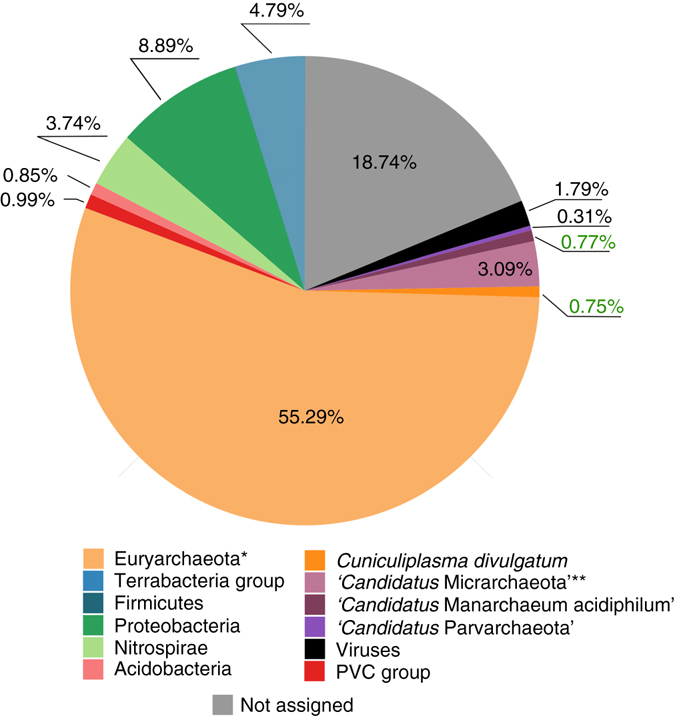
General structure of Parys Mountain acidic streamer community. Abundance values were calculated using median coverage of metagenomic contigs of each bin with normalisation to average genome size of bin representatives. Abundance values for *C. divulgatum* and ‘*Ca*. Mancarchaeum acidiphilum’ are highlighted with *green*. *excluding *C. divulgatum*. **excluding ‘*Ca*. Mancarchaeum acidiphilum’

Analysis of metagenomic contigs showed that the most abundant group (up to 57%) was Thermoplasmatales–related archaea. Small-genome archaeal lineages (‘*Candidatus* Parvarchaeota’ and ‘*Ca*. Micrarchaeota’) were also detected at 0.31% and 3.84%, respectively (Fig. 1).

**Fig. 2:**
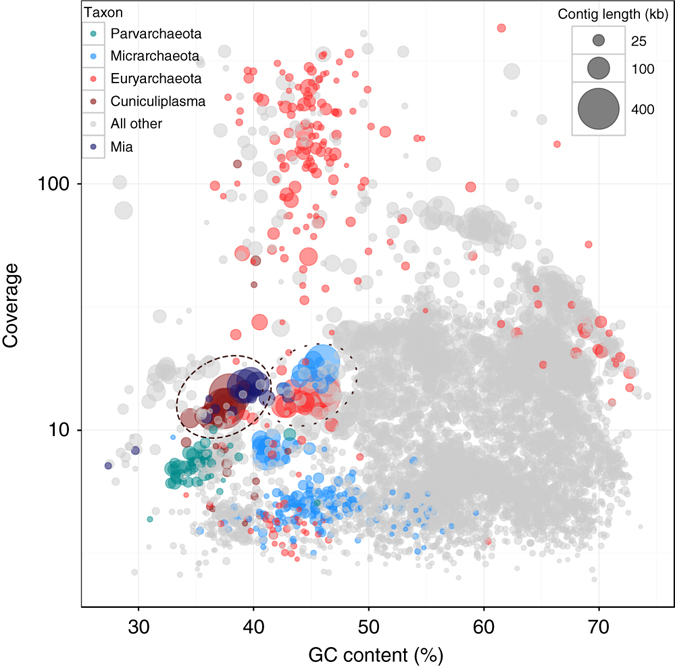
Distribution of Parys Mt. metagenomic contigs by coverage and GC content. Clusters of contigs related to *C. divulgatum*—‘*Ca*. Mancarchaeum’ and uncultivated Thermoplasmatales—‘*Ca*. Micrarchaeota’ excluding ‘*Ca*. Mancarchaeum acidiphilum’ microbial consortia are shown with *dotted-line ovals*

Interestingly, both median contig coverage and coverage-based abundance calculation indicate that the amount of *Cuniculiplasma* cells in the Parys Mountain acidic streamer is nearly equal to the amount of ‘*Candidatus* Mancarchaeum’ cells (Figs. 1 and 2). Also, analysis of read coverage vs. GC content of metagenomic contigs reveals that ‘*Ca*. Mancarchaeum’ and *C. divulgatum-*related contigs form a very compact cluster similar both in coverage and GC content (Fig. 2). Notably, we also observed another Thermoplasmatales, ‘*Ca*. Micrarchaeota’ contig cluster, in the Parys Mountain metagenome, suggesting that there are several stable two-member microbial associates in the community at this site.

### Fluorescence microscopy shows interaction of Mia14 and the host

**Fig. 3:**
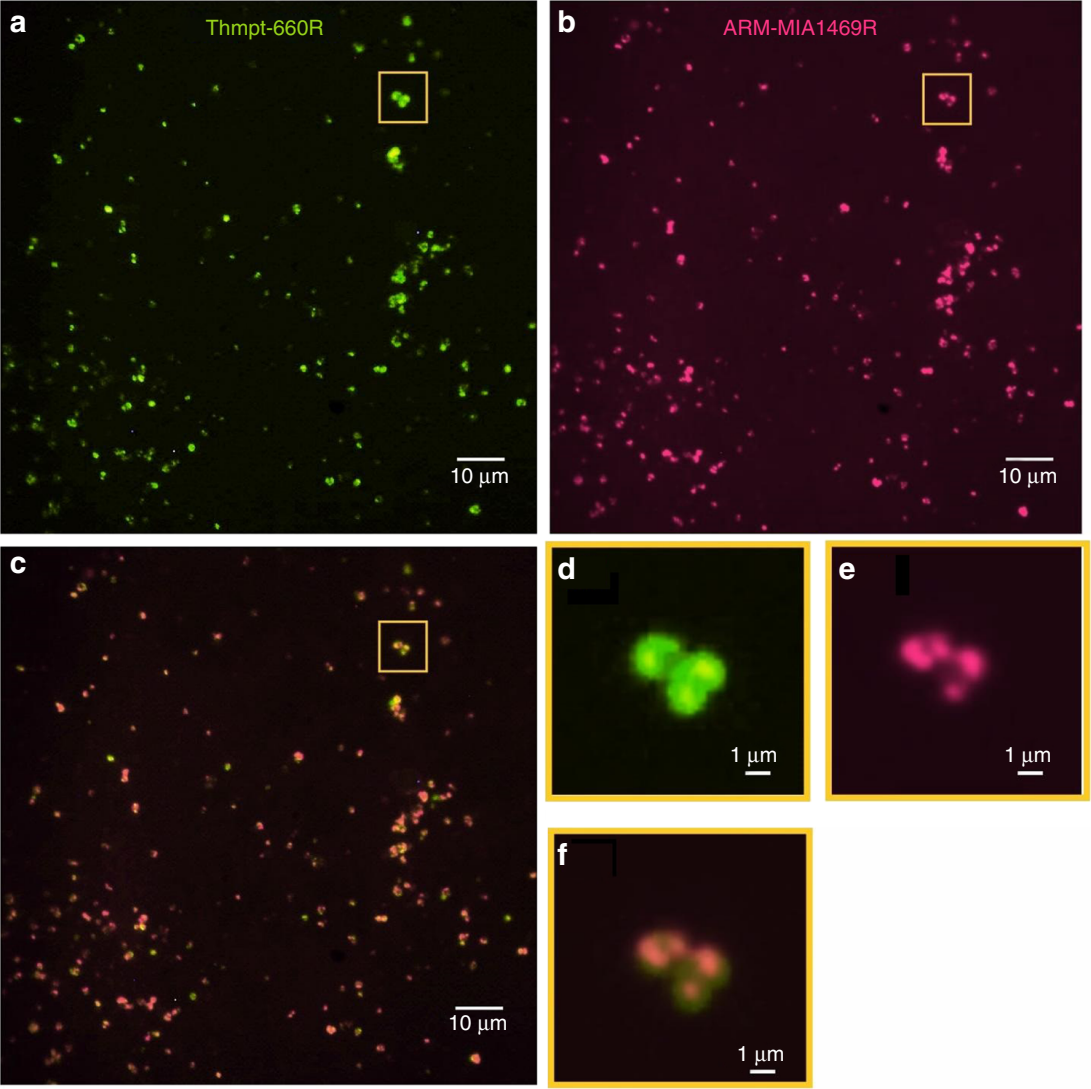
Archaeal cells visualised by CARD-FISH. Hybridisation with **a** probe Thpmt680R and **b** probe ARM-MIA1469R to target *Cuniculiplasma* spp. and ‘*Ca*. Mancarchaeum’, respectively. **c** Side-by-side comparison of ‘*Ca*. Mancarchaeum’ vs. *Cuniculiplasma*. ‘*Ca*. Mancarchaeum’ cells (*magenta*) localised on *green Cuniculiplasma* spp.). Panels **d**, **e** and **f** are the magnified images of *yellow-boxed* fields of panels **a**, **b** and **c**, respectively. The image was corrected with Daltonize tool (https://github.com/joergdietrich/daltonize) to improve perception of deuteranopic persons. Scale bars are 10 µm in panels **a**, **b** and **c** and 1 µm in panels **d**, **e** and **f**

Microbial cells from enrichment cultures set-up with the environmental sample from 2014 were either hybridised with probe EUB338(I-III) mix or probe ARCH915 to target Bacteria or Archaea, respectively. The following CARD-FISH analysis revealed dense populations of Archaea and the almost complete absence of bacterial cells. Pleomorphic morphologies of cells of various size, typical for *Cuniculiplasma*/Thermoplasmatales^16^, were confirmed with hybridisations with *Cuniculiplasma*-specific probe Clpm-1100R. Besides bright signals, the CARD-FISH microphotography retrieved numerous debris-like structural forms, which likely could be referred to as either dying or metabolically dormant cells. This observation is typical for both natural samples and initial enrichments, where the cells of different metabolic states coexist. Noteworthily, parallel hybridisations with ‘*Ca*. Mancarchaeum’-specific probe ARM-MIA1469R and Thermoplasmata-specific probe Thpmt680R showed quite similar images (Fig. 3), suggesting that the organisms live in a tight association. Cross-hybridisation of ARM-MIA1469R probe with pure *Cuniculiplasma* culture was controlled at specific hybridisation conditions and no positive signals were retrieved. Side-by-side comparisons of ‘*Ca*. Mancarchaeum’ vs. *Cuniculiplasma* cells revealed that the former are slightly smaller in size and only a minor fraction of cells do not overlap in each frame. Detailed view of some double-hybridised cell formations revealed single coccoid-shaped *Cuniculiplasma* cells were surrounded by ARM-MIA1469R probe-labelled organisms (Fig. 3f).

### Phylogenetic position of Mia14 and related organisms

Based on 16S ribosomal RNA (rRNA) gene sequence, Mia14 was found to be only distantly related to organisms with established taxonomic status. Less than 75 % SSU rRNA gene sequence identities with the thaumarchaeon *Nitrosospaera viennensis* and euryarchaeon *Methanosaeta consilii* are observed. Among candidate status holders (Supplementary Fig. 1), the nearest relative was inferred to be ARMAN-2 (‘*Candidatus* Micrarchaeum acidiphilum’^1, 5^) originally detected in acidic environments and sharing 92% 16S rRNA sequence identity with Mia14. Other similar sequences (92% sequence identity) belong to PCR-amplified and cloned SSU rRNA genes from fumarolic thermal and acidic green biofilms, Mexico, Michoacan, Los Azufres (KJ907762). Interestingly, both above sequences and the sequence of Mia14 possess introns in their 16S rRNA genes. In addition, sequences with a lower sequence identity and coverage (91%, 58%) were detected in a PCR-amplified SSU rRNA clone from Rio Tinto (FN865418)^18^, acidic hot springs (JF280243; 91%, 58%)^19^ and a number of other AMD and volcanic environments. Furthermore, similar sequences have been retrieved from southern Appalachian peatlands (PF82012)^20^ and wetlands in Finland (AM905392, AM905420)^21^. The two latter sites were oligotrophic, with temperatures in the range from 0 to 15 °C and slightly acidic pH (4–5.6 and 3.9–4.3, respectively). Along with wetland clones, similar signatures (BioProject PRJNA279923) have been found in metagenomic data from another oligotrophic environment, the pH-neutral groundwater from Fennoscandian terrestrial deep biosphere^22^. All these records suggest a wide distribution of organisms similar to Mia14 and ARMAN-2 in natural settings with various pH characteristics, not necessarily tied to acidic environments.

**Fig. 4:**
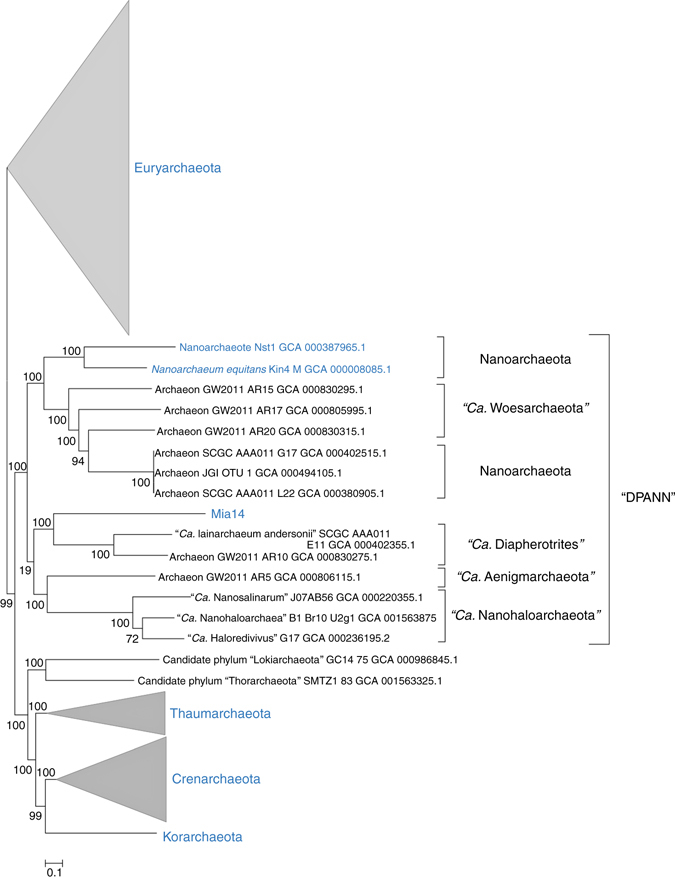
Phylogenetic position of Mia14 within *Archaea*. An approximate Maximum Likelihood tree based on the concatenated alignment of 56 ribosomal proteins universally conserved in *Archaea*. In total, 285 genomes were analysed. Taxa are named according to the NCBI taxonomy. Candidate phyla are shown in quotation marks. Lineages with cultured/co-cultured representatives are highlighted in *blue*. NCBI Genome Assembly IDs are shown for individual genomes. Scale bar reflects 0.1 substitutions per amino-acid position

The placement of Mia14 on the phylogenetic tree constructed with concatenated ribosomal proteins is presented in Fig. 4. In agreement with previous observations^12^, the position of Mia14 within the ‘DPANN’ superphylum is strongly supported.

### Genome statistics

**Fig. 5:**
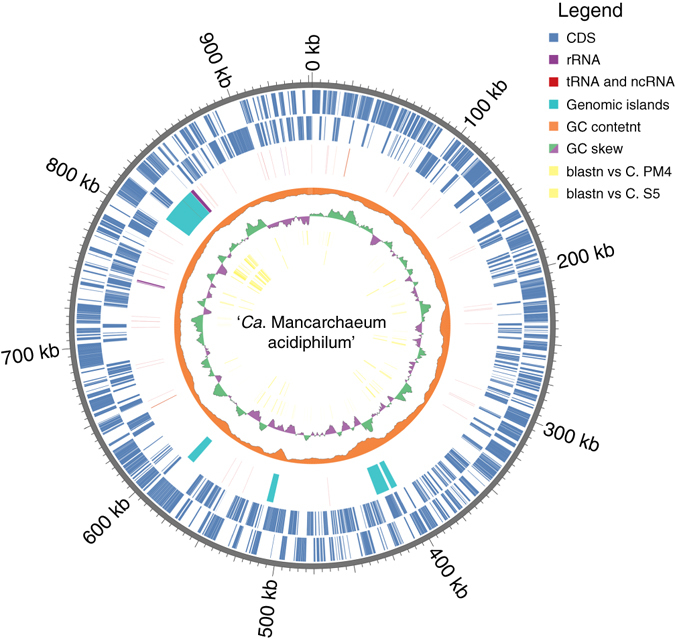
Genomic features and GIs in ‘*Ca*. Mancarchaeum acidiphilum’. *Rings* from outside to inside: genomic coordinates (*grey colour*); plus-strand CDS (*blue*); minus-strand CDS (*blue*); genomic islands (*green*) and RNA (*red*); GC-content (*orange*); GC-skew (*green*/*magenta*); blastn hits with e-value cutoff 10^−5^ vs. *C. divulgatum* PM4; blastn hits with e-value cutoff 10^−5^ vs. *C. divulgatum* S5

The genome of Mia14 is a single, circular chromosome with 952,257 bp, with the molar G+C% of 39.36% (Fig. 5). The coding density in the genome is of 1.032 genes per kbp (968 bases per gene). About ~ 150–200 hypothetical proteins were present. The genome encodes 45 transfer RNAs. Three introns were detected across the chromosome. All these traits are typical for small archaeal genomes, e.g., in *Nanoarchaeum equitans* (491 kbp)^23^, ‘*Candidatus* Nanobsidianus stetterii’, Nst1 belonging to the phylum Nanoarchaeota (592 kbp)^10^, ARMAN-2 (~ 1 Mbp)^5^ and other host-associated or symbiotic microorganisms.

### Lateral gene transfer between Thermoplasmatales and Mia14

Comparative analysis of in silico proteomes of Mia14 and strains S5 and PM4 with ProteinOrtho^24^ revealed several clusters of orthologous genes shared between Mia14 and *C. divulgatum* S5, but absent in *C. divulgatum* PM4 (Supplementary Data 1, Supplementary Fig. 2). These genes encode several membrane-associated proteins (MIA14_0876, _0886, _0893 and _0478), two SAM-dependent methyltransferases (MIA14_0883 and _0885), sulfocyanin (MIA14_0884) and peroxiredoxin (MIA14_0479). It should be noted that the majority of these proteins have homologues in PM4, but are more distant to those from both Mia14 and S5 and have different gene context. Few Mia14 genes from these clusters have no homologues in PM4.

Analysis with IslandViewer3^25^ showed that altogether five genomic islands (GIs) are present; the largest GI contains 41 genes and spans 36.5 kbp (Fig. 5). A closer inspection of this island reveals that the integration occurred in the gene for zinc-binding pyruvate-formate lyase-activating enzyme (MIA14_0850), splitting it in two parts: MIA14_0850 and MIA14_0891, with the latter located in the immediate vicinity of 23S rRNA gene. About 50% of genes within this GI could not be assigned to known arCOGs and represent small proteins that often contain transmembrane segments, which is typical for archaeal ‘dark matter’. In turn, the genes assigned to arCOGs (i.e., MIA14_0898, the DNA invertase Pin homolog, MIA14_0894 (similar to those from other Thermoplasmatales), ParA family chromosome partitioning ATPase and MIA14_0890, integrase of XerD family) were shown to be strongly associated with ‘dark matter’ islands in archaeal genomes and could be specifically attributed to integrated mobile elements^26^.

**Fig. 6:**
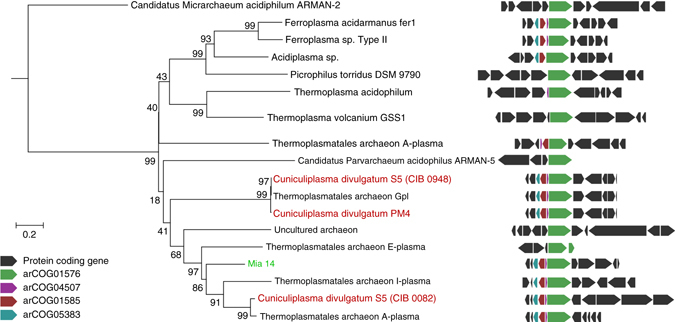
Phylogeny of MIA14_0877 and the neighbourhood of its gene. Gene neighbourhood of CDS for cation transport ATPase/copper-transporting P-type ATPase MIA14_0877 is shown on the right. Cation transport ATPases are shown in *green*, TRASH/YHS-like protein, metallochaperones (arCOG04507) are shown in *purple*, Lrp-AsnC family transcriptional regulators (arCOG01585) and conserved hypothetical proteins (arCOG05383) are shown in *cyan*. Other protein coding genes are shown as *dark grey* pentagons. Size of pentagons is proportional to size of corresponding proteins. The list of proteins included in the analysis with protein IDs is provided in Supplementary Table 1

Among GI-associated genes, we also found cation transport ATPase/copper-transporting P-type ATPase(MIA14_0877), which may have significance for the fitness of this organism in the harsh conditions of Parys Mountain AMD. Phylogenetic analysis of this ATPase showed that its close homologues are widely distributed among acidophilic Thermoplasmatales. At the same time, the copper-transporting ATPase of ARMAN-2 seems only quite distantly related to MIA14_0877 (Fig. 6, Supplementary Table 1). Gene neighbourhood of MIA14_0877 included an Lrp-AsnC family transcriptional regulator and a copper chaperone, resembling functional copper fitness islands described for ‘*Ferroplasma acidarmanus*’^27^. This gene cluster was found to be conserved in *Cuniculiplasma*-related archaea. Furthermore, *C. divulgatum* S5 genome possessed two copies of this copper-fitness island (Fig. 6). Interestingly, one of the *C. divulgatum* S5 copper fitness islands was adjacent to genetic loci for SHOCT family and DUF 302 family proteins as in the Mia14 copper gene cluster, while another *C. divulgatum* S5 copper gene island had a high level of gene synteny with *C. divulgatum* PM4 (Figs. 5 and 6). The above observation supports the lateral gene transfer from ancestral *Cuniculiplasma*-related lineage(s) to Mia14. In that case, it is more likely that LCA of *Cuniculiplasma* had two copies of this gene cluster, one of which was lost during the evolution of *C. divulgatum* PM4 and ‘G-plasma’.

Detailed analysis of de novo metagenome sequencing data from Parys Mountain samples shows that gene clusters similar to the abovementioned copper fitness island of Mia14 are widely present in different metagenomic contigs (Supplementary Data 2). It indicates that this highly mobile gene set is important for heavy metal resistance in microbial communities inhabiting acidic environments with high concentrations of dissolved metal ions.

Other smaller GIs of Mia14 (Fig. 5) contain defence systems (toxin/antitoxin and type III restriction-modification proteins), 2-oxoacid dehydrogenase multienzyme complexes, 2-oxoacid decarboxylase (E1) component subunits α and β, glycosyltransferases and numerous hypothetical proteins. Interestingly, the laminin G-encoding gene locus is also situated on the GI (see the section ‘Secretion systems’).

### Carbohydrate metabolism

The Mia14 genome has no genes for central carbohydrate metabolism pathways such as glycolysis and gluconeogenesis, pentose phosphate pathway or tricarboxylic acid (TCA) cycle. A detailed manual inspection suggested that the genome encodes a complete set of enzymes for glucose oxidation via the non-phosphorylating Entner-Doudoroff (ED) pathway^28^: a glucose dehydrogenase (MIA14_0575), d-gluconate dehydratase (MIA14_0298), 2-dehydro-3-phosphogluconate aldolase (MIA14_0299) and NAD-dependent d-glyceraldehyde dehydrogenase (MIA14_0297). Surprisingly, no enzymes for further conversion of glycerate, e.g., to glycerate-2-phosphate or glycerate-3-phopshate were found. The pyruvate released during the action of 2-dehydro-3-phosphogluconate aldolase, could be carboxylated to malate and oxaloacetate in the reaction catalysed by NAD-dependent malic enzyme (MIA14_0243/EC 1.1.1.38). Characterised homolog of *scf*A from *E. coli* (or *mae*A^29^) was reversible despite the carboxylation reaction being 28 times slower than the forward reaction. Other enzymes found to catalyse pyruvate conversions are phosphoenolpyruvate synthase/pyruvate phosphate dikinase (MIA14_0437, EC 2.7.9.2 and MIA14_0462) and pyruvate kinase (MIA14_0326, EC 2.7.1.40). It is worth mentioning that ARMAN-2, one of the most closely related organisms to Mia14 among those with partially sequenced genomes, exhibits a relatively scarce repertoire of genes in comparison to sibling lineages ARMAN-4 and -5. Only a few genes for glycolysis in ARMAN-2 and a near-complete set of genes in ARMAN-4 and -5 were predicted. TCA, which is dysfunctional in Mia14, was reported to be complete or almost complete in all ARMAN cluster organisms mentioned above^5^. Furthermore, central metabolic pathways in Mia14 starkly contrast with AR10 assembly representing ‘*Ca*. Diapherotrites’, but to some extent resemble those predicted for a more phylogenetically distant AR20 (‘*Ca*. Woesarchaeota’)^13^. Furthermore, the inspection of amino-acid biosynthetic pathways in Mia14 found them to be either incomplete or entirely missing. However, the total number of proteins in this functional category is higher in comparison to *N. equitans* and Nst1^10, 23^.

### Cofactors, vitamins, prosthetic groups and pigments

No genes for coenzyme A, folate, lipoic acid, NAD and NADP cofactor, pyridoxin (Vitamin B6), heme and siroheme, thiamin biosynthesis and riboflavin, FMN and FAD metabolism were present in the entire genome of Mia14. The lack of functional pathways for cofactors and amino acids is quite characteristic for organisms with reduced genomes^10, 23^.

### Protein metabolism

Protein processing and modification-related genes (G3E family of P-loop GTPases, peptide methionine sulfoxide reductase and Rio family of protein kinases, amino- and carboxy-terminal intein-mediated trans-splice, and ribonucleotide reductase of class III (anaerobic), large subunit (EC 1.17.4.2) were missing in Mia14. Altogether, we have identified 35 large- and 26 small-subunits of ribosomal proteins; L37E (arCOG04126), S17e (arCOG01885) and S27e (arCOG04108) were absent. All three and, correspondingly, two former proteins were found in *N. equitans* and Nst1 (Supplementary Data 3).

### Secretion systems

We have identified a number of genes affiliated with secretion processes in the genome of Mia14 (Supplementary Table 2). Two distinct type IV pili systems are present in the genome: one belongs to Methanococci/Methanothermobacteria/Thermococci group (MIA14_0170-_0177) and another is more similar to a euryarchaeal group (MIA14_0252-_0260)^30^. No archaellum-related genes were found, in agreement with the loss of motility in most of ‘DPANN’ species. Only one FlaK-like prepilin peptidase (MIA14_0570) was found. Key components of both systems are shared by many ‘*Ca*. Micrarchaea’ species. Additionally, the genome encodes Sec translocon genes for preprotein translocase subunits SecYE (MIA14_0832, _0132) and Sec61beta (MIA14_0736), SecDF (MIA14_0121 and_0122), signal peptide peptidase and signal recognition particle subunits and receptors. The presence of Sec-independent Tat pathway genes, suggests this system is operational for secretion of folded proteins.

The Mia14 surface layer deserves special attention. Besides a protection function in archaea, this compartment often regulates both cell adhesion and cell–cell interaction. We identified at least eight different proteins that eventually account for the architecture of the surface layer. It contains strain-specific secreted proteins with polycystic kidney disease (PKD) superfamily fold and β-propeller repeat domains fused to CARDB (cell adhesion related domain found in bacteria)-like adhesion module. Proteins of the β-propeller fold are ubiquitous in nature and widely used as structural scaffolds for ligand binding and enzymatic activity. This fold comprises between four and twelve four-stranded β-meanders, the so-called blades that are arranged circularly around a central funnel-shaped pore.

Another observation is the expansion of genes encoding jellyroll fold LamG-like proteins in the Mia14 genome, which are only distantly similar to other LamG proteins from archaea with *Candidatus* status and from bacteria, but generally abundant in ‘DPANN’ superphylum species. This finding suggests an association of these proteins with laminin (glycoprotein)-containing extracellular matrix and their key role in host cell interactions. Some of them are linked to aforementioned type IV pili loci and are localised in GI (Fig. 5).

### Respiration

The Mia14 genome encodes all typical subunits K, E, C, F, A, B, D, G, H and I of V/A Na^+^- and H^+^-transporting type ATP synthases, in this particular order (MIA14_0355-0364), whereas the genome of *N. equitans* encodes only five subunits of ATP synthase^23^. The analysis of conserved motifs supported H^+^-translocating V-type ATPase^31^.

All genes coding for cytochrome *bd* quinol oxidase were identified. Subunits I and II are encoded by MIA14_0653-0654. This type of oxidoreductase could generate proton motive force (PMF) by transmembrane charge separation, but do so without being a ‘proton pump’. The main electron acceptor for them is oxygen, but cytochrome *bd* oxidoreductases are usually induced in response to low oxygen concentrations and serve for oxygen detoxification^32^. The role of the cytochrome *bd* oxidoreductase in Mia14 is puzzling, as the organism completely lacks any genes for biosynthesis of isoprenoid quinones, which are the only electron donors for this electrogenic enzyme complex. Moreover, no genes were identified coding for electron donating type I NADH dehydrogenase or succinate dehydrogenase or other known respiratory complexes (III and IV).

### Transporters

ABC transporters, amino-acid permeases, Major Facilitator Superfamily and others have been predicted in Mia14 (Supplementary Table 3) to notably outnumber those in nanoarchaeal genomes^11^.

### Evolutionary patterns

**Fig. 7:**
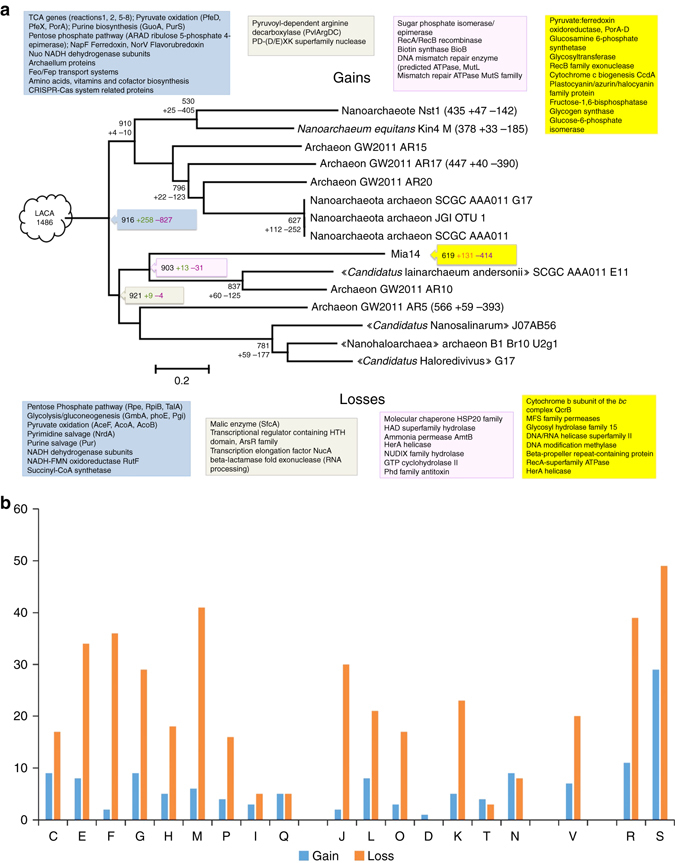
Gains and losses of arCOG families in the evolution of ‘DPANN’ group. **a** Reconstruction of gene loss and gain along the ‘DPANN’ subtree. Triplets of numbers indicate the estimates for the arCOG complement, arCOG gains and arCOG losses, respectively, for the selected extant or ancestral genomes and adjacent tree branches. Estimates for the terminal branches are shown next to the extant genome names. The number at the base of the tree indicates the arCOG complement with gains (+) and losses (−) estimated for the last archaeal common ancestor (LACA)^49^, ‘DPANN’ ancestor (*blue rectangle*), common ancestor of ‘*Ca*. Nanohaloarchaea’-‘*Ca*. Micrarchaea’ (*grey rectangle*), common ancestor of ‘*Ca*. Micrarchaea’ (*magenta rectangle*) and Mia14 (*yellow rectangle*). Losses and gains of selected protein families in course of evolution at above time-points are indicated in textboxes of same colours, with gains indicated in textboxes located above and losses listed in boxes below (see Supplementary Data 4 for further details). **b** Number of arCOGs predicted to be gained or lost in the course of evolution of Mia14 lineage with respective COUNT probability >50% by arCOG functional categories. The functional classification of the arCOGs is shown for two 4 major groups: C-Q—metabolic genes; J-N—informational genes; V—defence genes; R-S poorly characterised or uncharacterised genes (for details see ftp://ftp.ncbi.nih.gov/pub/wolf/COGs/arCOG/funclass.tab)

Overall, compared to other ‘DPANN’ group members, the Mia14 genome experienced an unusually high level of gene flux (Fig. 7). In addition to the 226 genes that do not belong to known arCOGs (a large fraction of such genes was probably also acquired at the terminal branches of the ‘DPANN’ tree), Mia14 has lost determinants for over 400 arCOG families from the genome of its common ancestor with ‘*Ca*. Iainarchaeum’/AR10 lineage (46% of the ancestral set), but also gained over 130 arCOGs (21% of its current arCOG complement). Gains and losses of comparable scale exist within the ‘DPANN’ group tree (e.g., the loss of 49% of the ancestral genome in the lineage of AR17 or acquisition of 18% of the gene complement in the lineage of G17-L22-OTU1), but not on the same tree branch. Gene gains and losses seem to affect all functional groups equally with a notable exception of the ‘Cell motility’ group where more gains than losses were predicted (Fig. 7). This functional group includes components of secretion systems, which might play a key role in interaction of Mia14 with its host. Moreover, as mentioned before many of the unique genes in Mia14 belong to GI, many of which encode membrane proteins and are associated with potential conjugative elements which might be involved in the extensive gene exchange between Mia14 and its host.

Analysing the trajectory of evolution of Mia14 from LACA through the prism of losses and gains of functional genes, a few interesting facts became apparent. Genes for the majority of enzymes of the TCA cycle were already lost during the transition from LACA to the ‘DPANN’ ancestor, together with many genes involved with amino acid, vitamin and cofactor biosynthesis along with the CRISPR-Cas system. Glycolysis and gluconeogenesis were present in all ascendants of Mia14 (i.e., in LACA, ‘DPANN’-and Mia14-‘*Ca*. Nanohaloarchaeota’-ancestors). However, many genes of these pathways were lost en route to the extant Mia14 species. Pathways for pyrimidine and purine biosynthesis and salvage were also lost at the very last (and long) step of evolution from Mia14/‘*Ca*. Ianarchaeum’ ancestor to the modern Mia14, with 414 genes lost and only 131 gained (Fig. 7a and Supplementary Data 4).

Analysis of the taxonomic affiliations of the ‘DPANN’ group species (Supplementary Data 5 and 6) shows that, in contrast with the other group members, the genome of Mia14 was and continues to be involved in extensive gene exchange with the Thermoplasmata lineages. Unsurprisingly, the most common source of the acquired genes is identified as *Cuniculiplasma divulgatum*, the Mia14 host.

### Etymology

‘*Candidatus* Mancarchaeum acidiphilum’

*Mancarchaeum* (*Manc.archaeum* M.L. mancus (adj.) crippled, maimed, referred to the absence of many pathways in the genome; N.L. neut. n. *archaeum* (from Gr. adj. *archaios* -*ê* -*on*, ancient), ancient one, archaeon; N.L. neut. n. Mancarchaeum, an archaeon with absence of many pathways in the genome.

a.ci.di’ phi.lum. M.L. neut. n. *acidum* from an acid; Gr. adj. philos from loving; M.L. neut. adj. acidiphilum means acid-loving.

## Discussion

In the present work, the enrichment culture from Parys Mountain AMD system was set-up to grow acidophilic members of the order Thermoplasmatales. The culture was eventually highly enriched in archaea from the genus *Cuniculiplasma*, and incidentally, with the significant (ca. 10% genomic reads or 20% of total population) community component belonging to yet uncultured archaea distantly related with ARMAN-2. Due to its high numbers in the enrichment, we were able to produce the fully assembled genome of the ‘ARMAN’-related organism. Based on the genome annotation and experimental data (co-existence in an enrichment culture and fluorescence microscopy), we inferred that the metabolic needs of this sentinel of *Cuniculiplasma* spp. termed ‘*Ca*. Mancarchaeum acidiphilum’ resemble to some extent those of other archaea co-occupying the environment (e.g., reliance on external proteinaceous compounds and amino acids). However, the incompleteness or absence of the central metabolic pathways (e.g., TCA, glycolysis, quinone biosynthesis, etc.) and reduced genome size support an obligate partner-dependent (or ‘ectoparasitic’) lifestyle. Our data (Fig. 3) further suggest that sizes of Mia14 cells (and likely other ARMAN-related archaea) have a broad range, usually larger than the diameter of membrane filter pores (0.22 μm) used to enrich for these organisms. The penetration of cells through the 0.22 μm pores of membrane filters observed previously^3^ may also be explained by the lack of rigid cell walls in these organisms. For example, the majority of 1–2 µm, cell wall-deficient Thermoplasmatales may squeeze through pores of this diameter.

The occurrence of laterally transferred genes and GIs from *Cuniculiplasma* spp. in Mia14 highlights the relative connection between these organisms co-existing in one environment. It is, furthermore, likely that extracellular structures such as pili or pili-like organelles might be present in Mia14. One may also speculate on massive exchange of DNA through some cell pores or by using the Type IV pili system and numerous membrane proteins encoded within GIs, the likely conjugative elements.

Under our experimental conditions, the preferred partner of Mia14 was *Cuniculiplasma divulgatum* (previously known as ‘G-plasma’^16^), which is an abundant inhabitant in AMD. However, the distribution of archaea related to Mia14 (or to ARMAN-2 cluster) in diverse, sometimes non-acidic environments, emphasises their higher plasticity and ability to adapt to the broader range of environmental conditions. This broader distribution of ‘ARMAN’-related organisms in other environments also suggests that *Cuniculiplasma* spp. may not necessarily be the exclusive partner (host) for ARMAN-2-like organisms.

Mia14 is characterised by a very rudimentary metabolic capability. It is even devoid of minimal sets of enzymes required for biosynthesis of both types of nucleotides (purine and pyrimidine) and of 12 out of 20 amino acids (lysine, methionine, arginine, asparagine, alanine, aspartate, leucine, isoleucine, threonine, phenylalanine, tyrosine and tryptophan). Biosynthetic pathways for vitamins and cofactors (B1, B2, Coenzyme A, Coenzyme PQQ, B6, B12, heme, methanopterin, andubiquinone/menaquinone) are incomplete.

In Mia14, all glycolytic enzymes are missing. The majority of enzymes for the pentose-phosphate pathway and the entire TCA cycle are also absent. On the other hand, the non-phosphorylating ED pathway of glucose oxidation is present. Additionally, fatty acid metabolism and beta-oxidation, folate cycle, phospholipid biosynthesis, aminosugar metabolism, glycine and serine catabolism pathway, urea cycle and amino group metabolism, nicotinamide, pyruvate metabolism and interconversion of pyruvate and acetyl-CoA, trehalose biosynthesis, glycogen metabolism and biosynthesis, propionate metabolism, heme biosynthesis, pentose-phosphate pathway (non-oxidative phase) and lipopolysaccha﻿rides (LPS) synthesis are absent. Furthermore, we have not found any substrate-level phosphorylation pathways. The Mia14 respiratory chain is also absent; no Complex I (NADH:ubiquinone oxidoreductase)^33^, Complex II (succinate:quinone oxidoreductase)^34^, Complex III (either cytochrome *bc*_*1*_ complex^35^ or ACIII^36^) or Complex IV (heme-copper oxygen reductases)^37^ proteins-coding genes were found in the genome. However, the presence of H^+^-translocating V-type ATP synthase in the organism suggests the activity of PMF-generating complexes. The only candidate complex for this role is the cytochrome *bd* quinol oxidase, which was found in the genome. The lack of appropriate endogenous electron donors for this complex in Mia14, which is deficient in isoprenoid quinone biosynthesis, could be compensated by exogenous quinones from the membrane of *Cuniculiplasma* sp., considering the assumption of mutualistic interactions between Mia14 and this organism. Indeed, the QH_2_ oxidising cytochrome *b*_*558*_ in the Mia14 cytochrome *bd* complex is localised on the surface of the cell membrane, as inferred from topology prediction and alignment of MIA14_0653 amino-acid sequence with its extensively characterised homolog from *E. coli* ^31^. As both *Cuniculiplasma* species^15^ lack cell walls and their cells are usually found in tight contact with Mia14 (Fig. 3), we can speculate that the latter organism utilises a broad diversity of *Cuniculiplasma* membrane quinones (either from living or dead cells) as electron donors for energy conservation. However, no genes of canonical heme biosynthesis, heme import pathways^38^ or an alternative pathway for the formation of heme^39^ have been found in the Mia14 genome.

Besides the possibility of a completely novel heme biosynthesis pathway in this archaeon, the only way for proper assembly of the cytochrome *bd* complex is the incorporation of exogenous hemes. Accumulation of exogenous hemes in the membrane, which is capable of complementing the growth of heme-deficient organisms, has been demonstrated for pathogenic bacteria^40^. Considering that hemes *b* and *d* bind covalently to apoproteins and that the heme-binding amino acids are localised close to the surface of the cell membrane in cytochrome *bd* complexes^31^, it seems possible for Mia14 to acquire exogenous hemes from *Cuniculiplasma* spp. to assemble its only PMF-generating complex. It should be noted that the complete set of genes for canonical or non-canonical heme biosynthesis pathways is also absent in *Cuniculiplasma* strains PM4 and S5, although these aerobically respiring organisms possess heme-containing enzymes of the electron transfer chain^16^. It, therefore, seems possible that *Cuniculiplasma*, and probably Mia14, possess yet unknown mechanisms of heme biosynthesis.

In many archaea, the surface layer is the only cell envelope component providing all functions normally associated with a cell wall, i.e., acting as the protective barrier and maintaining the cell shape. However, in some cases the surface layer proteins may also help in cell–cell association^41, 42^. The Mia14 surface layer likely possesses a very complex and unique architecture, consisting of at least eight strain-specific secreted surface proteins. It is noteworthy that only four of these surface proteins (MIA14_0152, _0331, _0793 and_0946) require almost 2.5% of the whole genome. We identified two domain types in surface layer proteins displaying the PKD superfamily fold and beta-propeller Kelch and YVTN β-repeat domains fused to CARDB (cell adhesion related domain found in bacteria)-like adhesion module. Six of these surface layer proteins are predicted to be gained from various methanogenic and acidophilic euryarchaea and the members of ‘TACK’ superphylum. As previously hypothesised^42^, the expansion of proteins containing PKD and YVTN domains indicates their function in cell–cell interactions. Thus, we propose that the very rudimentary metabolic capability of Mia14 indicates a *Cuniculiplasma-*associated lifestyle and that numerous systems such as type IV pili, surface proteins and membrane channels provide an interface for the exchange of metabolites, energy, macromolecules including DNA between Mia14 and its host.

## Methods

### Sample proceedings

Samples from sediments and water of acidic streamer were taken for the establishment of enrichment cultures in March of 2011 from copper-containing sulfidic ores, Parys Mountain, Anglesey, North Wales, UK (53°23′13.6′′N 4°20′58.6′′W). The enrichment cultures were supplemented with yeast extract and glucose each at concentrations of 0.1% (w/vol) and grown at pH 1–1.2 and 37 °C in AB medium^15^.

DNA was extracted by G’NOME DNA Kit (MP Biomedicals). For the metagenomic study and second series of enrichment cultures set-up for CARD-FISH experiments, sediments and water were collected in July, 2014 from the same sampling spot as in March, 2011. The metagenomic DNA was isolated with DNA Power Isolation Kit for Soil (MoBio).

DNA concentrations in all cases were measured using Varian Cary Eclipse fluorescence spectrophotometer using Quant-iT DNA Assay Broad Range Kit (Life Technologies).

### Genome sequencing and annotation

The genomes were sequenced, assembled and annotated at Fidelity Systems, Inc. (Gaithersburg, MD), as previously reported^16^. Final assemblies provided *ca*. 564 and 561-fold coverages for strain S5 and PM4, respectively^16^, while Mia14 genome was covered 42-fold. GIs were inspected using Island Viewer 3^25^ using two different algorithms: IslandPath-DIMOB, based on the analysis of mobile element-related genes and dinucleotide distribution biases^43^, and SIGI-HMM, exploiting biases of codon usage implementing a hidden Markov model approach^44^. In most cases, after manual inspection of taxonomic affiliation of best blast hits of predicted horizontally transferred proteins, both predictions were considered as GIs. Analysis of proteins shared between Mia14 and *C. divulgatum* S5, but absent in *C. divulgatum* PM4, was performed by ProteinOrtho^23^ V5.15 using default parameters (10^−5^ blastp e-value, 50% minimal query coverage and 25% minimal percent identity).

Based on the genomic data, the specific primers for the detection of Mia14-related organisms in enrichment cultures were: 5′—3′ F Micr (GCTTGGCGAATAAGTGCTGGGC) and R Micr (ATCTTGCGACCGTACTCCCCAG).

### Metagenome sequencing of Parys Mountain community

For sequencing of the Parys Mountain acidic streamer metagenome, both paired-end and mate-paired DNA libraries were used. Paired end library was prepared from 400 ng of enviromental DNA with NEBNext Ultra DNA library preparation kit (New England Biolabs, Ipswich, USA) according to the manufacturer’s instructions to obtain mean library size of 500 bp. Mate-paired libraries were prepared with Nextera Mate Pair Library Prep Kit (Illumina Inc., San Diego, CA, USA) using gel-free protocol supplied by manufacturer. Both libraries were sequenced with 2 × 250 bp reads with MiSeq Personal Sequencing System (Illumina Inc., San Diego, CA, USA). After sequencing, all reads were subjected to stringent quality filtering with CLC Genomics Workbench 8.5 (Qiagen, Germany). After filtering, overlapping paired-end library reads were merged with SeqPrep tool (https://github.com/jstjohn/SeqPrep) resulting in 4,110,617 single reads and 7,539,176 read pairs. Mate paired reads were treated with NextClip tool^45^, resulting in 663,171 read pairs with mean insert size of 2170 bp. Reads were assembled with metaSPADES^46^, resulting in metagenomic assembly of about 200 Mb of total length consisting of 93,342 contigs with N50 of 3295.

For the binning for metagenomic contigs they were aligned against NCBI non-redundant protein database using DIAMOND in ‘blastx’ mode^47^ with e-value of 10^−6^. Results of the alignment were imported to MEGAN 6.4.22^48^ with default settings adjusted as follows: min score—80, top percent—10, min support—20. Binning by MEGAN was performed using default settings. After the initial automatic binning step, additional manual inspection was performed. In particular, contigs with ambiguous taxonomic affiliation, characterised by mixed blastx hits were either reassigned to a bin of a higher taxonomic level or moved to the ‘Unassigned’ bin. *Cuniculiplasma sp.*—related and Mia14-related contigs were identified manually using blasting their genome sequences with blastn against the local Parys Mountain metagenomic contigs nucleotide blast database.

For the calculation of the taxon abundance, all metagenomic reads were mapped to the contigs with Bowtie 2^49^. Total length of all sequencing reads mapped to every particular bin was calculated with samtools^50^. Abundance was calculated as a ratio between total lengths of all sequencing reads to the average genome size of the corresponding taxon (based on NCBI genomes database). Relative abundance value which was used for the Fig. 1 was calculated as ratio of bin abundance to the sum of bin abundances.

### CARD-FISH

Samples were fixed for 1 h at room temperature with pre-filtered formaldehyde (final concentration 2% vol/vol). Sample (diluted from 10^−1^ to 10^−3^, according to cell concentrations) was filtered through 0.22 μm (Ø 25 mm) polycarbonate membranes (New Technologies Group Srl, NTG). Cell permeabilisation was performed by incubation for 1 h with lysozyme (10 mg ml^−1^ in TE buffer pH 8.0) followed by incubation with a chromopeptidase for 30 min (5 mg ml^−1^), both at 37 °C. Filters were cut into sections and cells were hybridised with universal horseradish peroxidase (HRP)-labelled oligonucleotide probes for *Eubacteria* (EUB338 I, II, III probe mix)^51, 52^ to check for bacterial presence and for *Archaea* (Arch915)^53^. Absence of unspecific hybridisation was controlled by implication of the nonspecific probe NON338. The CARD-FISH probes specific for members of order Thermoplasmatales (Thpmt-680R), of family Cuniculiplasmataceae (Clpm-1100R) and of ‘*Ca*. Mancarchaeum acidiphilum’ Mia14 (ARM-MIA1469R) were designed through this study. Detailed information about the probes is given in Supplementary Table 4. Intracellular peroxidase was inhibited by treatment with 1% H_2_O_2_ at room temperature for 20 min. For signal amplification tyramide-Alexa488 and -Alexa594 were used^54^. The filter sections were counter-stained with 4′,6-diamidino-2-phenylindole (DAPI) (2 μg ml^−1^) in a four-to-one ratio of Citifluor (Citifluor Ltd, Leicester, UK): Vectashield (Linaris GmbH, Wertheim-Bettingen, Germany). At least 200 DAPI-stained and Alexa-positive cells were counted in a minimum of 10 fields under an AXIOPLAN 2 Imaging microscope (Zeiss, Germany).

### Sequence analysis and evolutionary reconstructions

Protein coding genes of Mia14 were assigned to archaeal Clusters of Orthologous Groups (arCOGs) as follows: PSSMs derived from arCOG alignments were used as PSI-BLAST queries in a search against a database of archaeal proteins with e-value cutoff of 10^−4^. Proteins (fragments) were assigned to arCOGs with the highest-scoring hits^55^. Also, sequences of the 56 ribosomal proteins universally conserved in archaea^56^ from 285 organisms with completely or almost completely sequenced genomes were aligned using the MUSCLE program^57^. Alignments were concatenated; the phylogenetic tree was reconstructed using the FastTree program^58^ with WAG evolutionary model and gamma-distributed site rates.

Manual curation of automatic functional predictions was performed according to the recent protocol^59^. In particular, the proteins of central carbohydrate pathways (Embden-Meyerhoff and Gluconeogenesis, ED, pentose-phosphate, TCA) including currently known archaeal modifications^60^ were searched by BLAST (with a consciously low e-value cut-off = 1.0 to avoid loss of distantly related sequences) of sets, including several amino-acid sequences of biochemically characterised (mainly, that with ‘Evidence at protein level’ in Swissprot database) archaeal and bacterial proteins against the genome (tBLASTn) or in silico translated proteome (BLASTp) of Mia14. If no hits with all queries were found the protein was regarded as absent. If all/many of proteins of the pathway were absent the pathway was regarded as absent. If any BLAST hits were obtained, these sequences were BLASTed against Uniprot and Swissprot (e-value threshold = 0.01) and resulted hits analysed. The co-localisation of genes for a particular pathway was also taken into account.

arCOG phyletic patterns of the 15 ‘DPANN’ group genomes were analysed using the COUNT program^61^ as described previously^62^. A matrix with the numbers of orthologs in the given arCOG in the given organism and the tree of the corresponding genomes were used to estimate the parameters of a phylogenetic birth and death model with gamma-distributed gain, loss and duplication rates^61^. The solution produces posterior probabilities for the presence or absence of a gene in ancestral genomes as well as the probabilities of gene gains and losses on all tree branches, providing a comprehensive picture of these events in the evolutionary history of the ‘DPANN’ group. The reconstructed ‘DPANN’ group ancestor was compared to the previously reconstructed last common ancestor of all *Archaea* ^63^.To identify actual arCOGs in three groups (likely present, lost, gained) probability of each event more or equal 50% has been chosen for each lineage of interest.

Taxonomic affiliations for proteins, encoded in the 10 out of 15 ‘DPANN’ genomes (to the exclusion of the three genomes in the Nanoarchaeota archaeon SCGC AAA011-G17 lineage and two genomes in the ‘*Candidatus* Haloredivivus’ lineage that have close relatives within the ‘DPANN’ group) was assessed by running protein BLAST search other archaeal genomes. The database contained 704,591 proteins from 286 complete and nearly complete archaeal genomes, available at GenBank and the protein set encoded by Mia14. The top BLAST hit (e-value threshold of 10^−6^) outside of the self genome was recorded as an approximate indication of the taxonomic affiliation of the protein.

### Data availability

Sequence data determined in this study are available at NCBI under BioProject Accession PRJNA353339. Genome sequencing and assembly are deposited in the GenBank under Accession CP019964. Metagenomic reads and contigs were submitted to MG-RAST and can be provided from the corresponding author upon request.

## Electronic supplementary material

Supplementary Information

Supplementary Data set 1

Supplementary Data set 2

Supplementary Data set 3

Supplementary Data set 4

Supplementary Data set 5

Supplementary Data set 6

Peer Review File
