# Supplementary material for: ‘ARMAN’ archaea depend on association with euryarchaeal host in culture and in situ
Source: Nat Commun. 2017 Jul 5;8:60. doi: 10.1038/s41467-017-00104-7 (PMC5498576; doi:10.1038/s41467-017-00104-7)
Supplement: Supplementary file 1 — Supplementary Information [file 41467_2017_104_MOESM1_ESM.pdf]

File name: Supplementary Information
Description: Supplementary Figures, Supplementary Tables and Supplementary References

File name: Supplementary Data 1
Description: Groups of similar proteins identified in *C. divulgatum*-related and "*Ca.* Mancarchaeum acidiphilum" genomes.

File name: Supplementary Data 2
Description: Detailed analysis of de novo metagenome sequencing assemblies from Parys Mountain showed that gene clusters similar to abovementioned copper fitness island of MIA14 are widely presented in different metagenomic contigs.

File name: Supplementary Data 3
Description: 218 archaeal core genes (arCOGs) in Mia14 and two nanoarchaeal genomes.

File name: Supplementary Data 4
Description: Inference of gene presence for four lineages along the tree of DPANN group leading to Mia14.

File name: Supplementary Data 5
Description: arCOGs inferred to be lost in Mia14 lineage.

File name: Supplementary Data 6
Description: Breakdown of best BLASTP hits for Mia14 proteins against all archaea in the phylogenetic tree (Fig. 4) plus two *Cuniculiplasmas genomes.*

File name: Peer Review File
Description:

Supplementary Information

Supplementary Table 1. List of Cation transport ATPases included in the phylogenetic analysis

Organism	Protein ID
"*Candidatus* Micrarchaeum acidiphilum" ARMAN-2	EET90099.1
"*Ferroplasma acidarmanus*" fer1	AGO61360.1
Acidiplasma sp. MBA-1	KJE49923.1
Acidiplasma aeolicum	KPV45991.1
Acidiplasma cupricumulans	KQB34972.1
Picrophilus torridus DSM 9790	AAT42749.1
Thermoplasma acidophilum	CAC12269.1
Thermoplasma volcanium GSS1	BAB60406.1
Thermoplasmatales archaeon "A-plasma"	EQB69441.1
"*Candidatus* Parvarchaeum acidophilus" ARMAN-5	EFD92740.1
Cuniculiplasma divulgatum S5	CSP5_0948
Thermoplasmatales archaeon "G-plasma"	EQB69951.1
Cuniculiplasma divulgatum PM4	CPM_0944
uncultured archaeon, identified by metagenome sequencing of acid sulfate soils from South Australia (Bioproject PRJNA256113_	AKA49370.1
Thermoplasmatales archaeon "E-plasma"	EQB66611.1
"*Ca*. Mancarchaeum acidiphilum"	Mia14 0877
Thermoplasmatales archaeon "I-plasma"	EQB66294.1
Cuniculiplasma divulgatum S5	CSP5_0082
Thermoplasmatales archaeon "A-plasma"	EQB70551.1

Supplementary Table 2. Type IV pili system genes in Mia14 genome

MIA14_ 0118	Pilin/Flagellin, contains class III signal peptide
MIA14_ 0152	Surface protein possibly associated with type IV pili like system
MIA14_ 0170	Pilus assembly protein, ATPase of CpaF family
MIA14_ 0172	Pilus assembly protein TadC
MIA14_ 0173	Pilus assembly protein TadC
MIA14_ 0176	Secreted protein, component of type IV pili like system
MIA14_ 0194	Surface protein, possibly associated with type IV pili like system
MIA14_ 0252	ATPase involved in archaellum/pili biosynthesis
MIA14_ 0253	Pilus assembly protein TadC
MIA14_ 0253	Component of type IV pili system
MIA14_ 0254	Pilin/Flagellin, contains class III signal peptide
MIA14_ 0255	Pilin/Flagellin, contains class III signal peptide
MIA14_ 0256	Pilin/Flagellin, contains class III signal peptide
MIA14_ 0257	Pilin/Flagellin, contains class III signal peptide
MIA14_ 0258	Pilin/Flagellin, contains class III signal peptide
MIA14_ 0259	Component of type IV pili system, contains class III signal peptide
MIA14_ 0260	Pilin/Flagellin, contains class III signal peptide
MIA14_ 0342	S-layer protein, possibly associated with type IV pili like system
MIA14_ 0570	Peptidase A24A, prepilin type IV
MIA14_ 0572	CARDB cell adhesion domain containing protein
MIA14_ 0940	Pilus assembly protein, ATPase of CpaF family
MIA14_ 0946	Surface protein, possibly associated with type IV pili like system

Supplementary Table 3. Transporters in Mia14 genome

MIA14_ 0064	ABC-type multidrug transport system, permease component
MIA14_ 0161	DMT superfamily transporter
MIA14_ 0162	DMT superfamily transporter
MIA14_ 0187	MFS family permease
MIA14_ 0197	ABC-type multidrug transport system, ATPase component
MIA14_ 0198	ABC-type multidrug transport system, permease component
MIA14_ 0305	Predicted transporter component, contains two sulfur transport domains
MIA14_ 0341	ABC-type antimicrobial peptide transport system, ATPase component
MIA14_ 0343	ABC-type antimicrobial peptide transport system, permease component
MIA14_ 0389	MFS family permease
MIA14_ 0393	Amino acid transporter
MIA14_ 0395	MFS family permease
MIA14_ 0397	MFS family permease
MIA14_ 0416	MFS family permease
MIA14_ 0426	Permease of the drug/metabolite transporter (DMT) superfamily
MIA14_ 0429	MFS family permease
MIA14_ 0560	Predicted metal permease
MIA14_ 0582	Amino acid transporter
MIA14_ 0583	Ammonia permease
MIA14_ 0584	Cation transport ATPase
MIA14_ 0591	Amino acid transporter
MIA14_ 0655	ABC-type multidrug transport system, ATPase component
MIA14_ 0656	ABC-type multidrug transport system, permease component
MIA14_ 0693	MFS family permease

MIA14_ 0695	MFS family permease
MIA14_ 0720	MFS family permease
MIA14_ 0734	Membrane protein, possible transporter
MIA14_ 0760	Sulfite exporter, TauE/SafE family
MIA14_ 0766	MFS family permease
MIA14_ 0768	Predicted Fe2+/Mn2+ transporter, VIT1/CCC1 family
MIA14_ 0814	Amino acid transporter
MIA14_ 0888	Co/Zn/Cd efflux system component
MIA14_ 0911	MFS family permease
MIA14_ 0985	ABC-type Na+ efflux pump, permease component
MIA14_ 1014	ABC-type multidrug transport system, ATPase component

Supplementary Table 4. CARD-FISH probes and cell dimensions recognised in this study

**Probe**	**Sequence**	**T_{hybr}**[a], °C	**T_{wash}**[b], °C	**FA**[c] **(%)**	**Cell dimensions (µm)**
Thmpt-680R	GGATTACAGGATTTTACCCCT	37	38	20	0.5 - 1.8
Clpm-1100R	CTTCAAAGTTCTACCAAC	46	48	0	0.5 – 1.8
ARM-MIA1469R	TTGAGGTGATCTATCCGCAGG	46	48	20	0.4 - 1.2

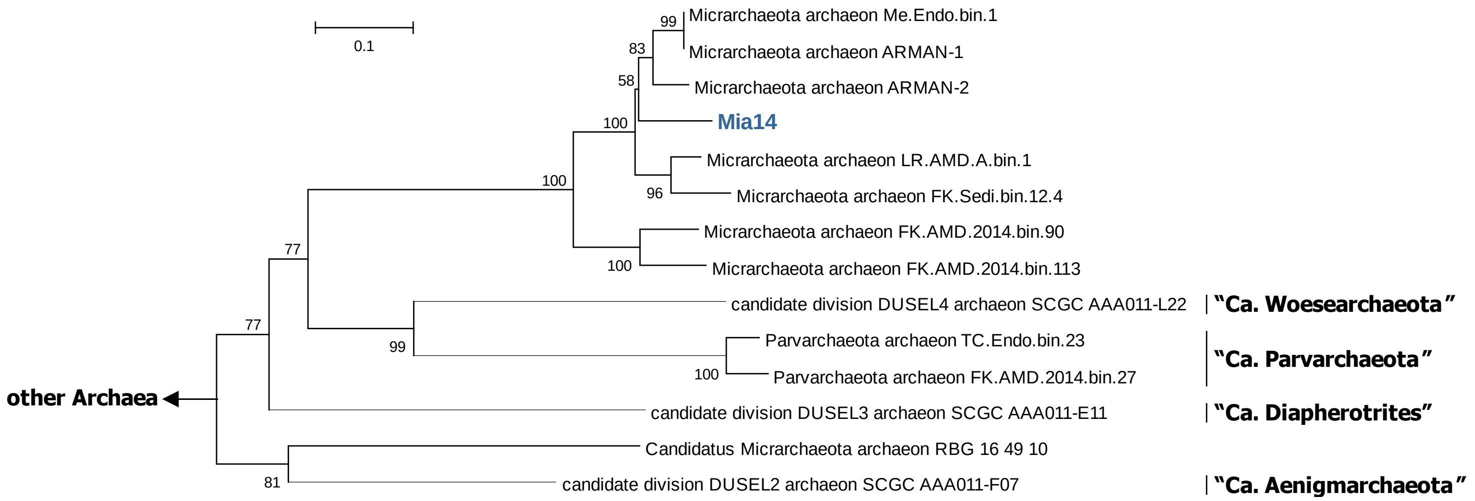

Supplementary Figure 1. SSU rRNA phylogeny of Mia14 and closely related organisms of "DPANN" superphylum. Maximum Likelihood tree constructed using methodology described earlier[1]. The analysis involved 150 nucleotide sequences. There were a total of 1264 positions in the final dataset. Scale bar represents 0.1 substitution per nucleotide position.

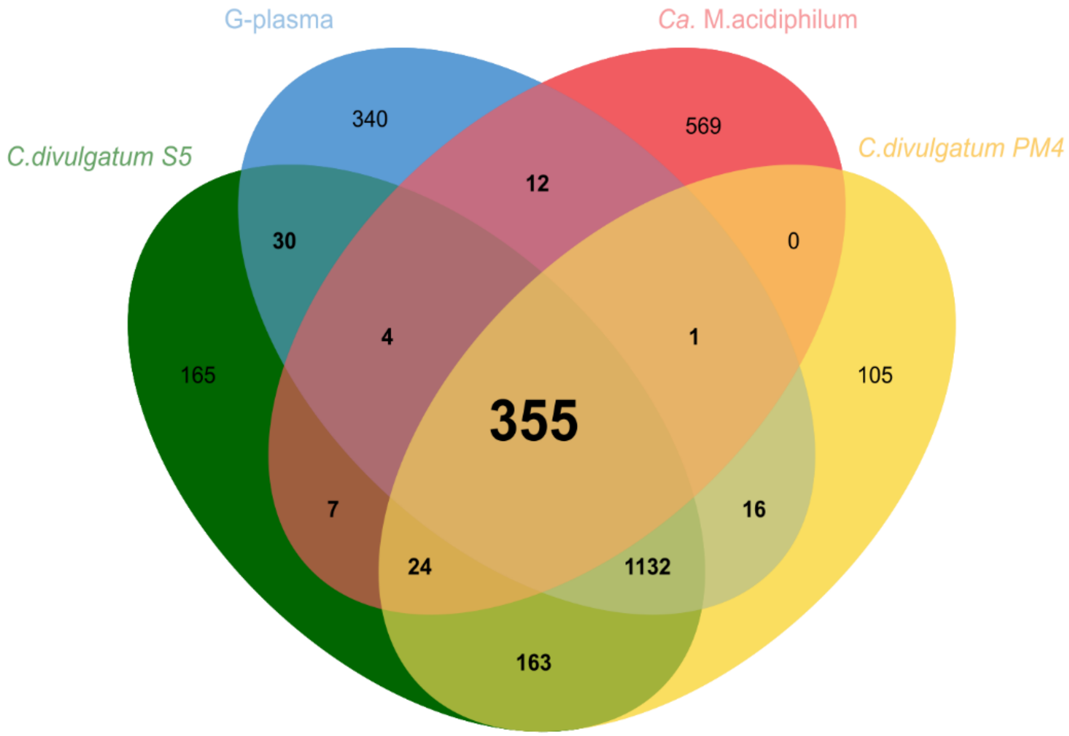

Supplementary Figure 2. Groups of similar proteins in *C. divulgatum*-related and "*Ca.* Mancarchaeum acidiphilum" genomes. Protein groups were identified with ProteinOrtho V5.15 (ref. [2]) with default parameters.

Supplementary References

1. Golyshina, O. V. *et al.* The novel, extremely acidophilic, cell wall-deficient archaeon *Cuniculiplasma divulgatum* gen. nov., sp. nov. represents a new family of *Cuniculiplasmataceae* fam. nov., *order Thermoplasmatales. Int. J. Syst. Evol. Microbiol.* **66,** 332-340 (2016).

2. Lechner, M. *et al.* Proteinortho: detection of (co-)orthologs in large-scale analysis. *BMC Bioinformatics* **12**, 124 (2011).
