# Supplementary material for: ‘ARMAN’ archaea depend on association with euryarchaeal host in culture and in situ
Source: Nat Commun. 2017 Jul 5;8:60. doi: 10.1038/s41467-017-00104-7 (PMC5498576; doi:10.1038/s41467-017-00104-7)
Supplement: Supplementary file 8 — Peer Review File [file 41467_2017_104_MOESM8_ESM.pdf]

Reviewers' comments:

Reviewer #1 (Remarks to the Author):

Golyshina et al obtain a coculture of ARMAN belonging to Micrarchaeota phylum with that of member of the Thermoplasmates, using microscopy they show that they are likely in contact with one another as has been shown in nature. From this culture a complete genome of the ARMAN was obtained and used to look at gaps in metabolisms and gene transfer. Overall I found the paper to be fairly well written, a bit over wordy at times. The discussion about missing genes is relevant and interesting given the subject. However, many of the findings are similar to what has been seen in other DPANN, and they didn't not cite this work, Castelle et al. 2015 Current Biology. I think that this does advance our understanding of DPANN archaea, I just think the findings need to be placed in better context of what has been shown recently. There also needs to be a more robust sampling of genomes included in the phylogenetic analyses.

My other big issue is that few genomes (Woesearchaeota, Pacearchaeota and others from Castelle) were included in the analyses to look at gene transfer. If you are going to say that ARMAN has had more LGT that other DPANN I think the full set of available genomes should be included. This is really interest analyses, however.

I find the title difficult to read and interrupt.

Why are Loki and Thor shown in bold in figure 4? These are not even discussed in the text.

The individual DPANN phyla should be labeled in this tree, fig 4. Especially which MIa44 belongs to! It is Micrarchaeota or Parvarchaeota? Also this tree lacks other ARMAN genomes, that is a must! This tree is actually missing lots of DPANN, but it needs to include other ARMAN genomes at least.

It's phylogenetic position in the DPANN phyla, which phylum, should clearly stated in the text as well.

Reviewer #2 (Remarks to the Author):

The paper by Golyshina et al report on the first ungapped genome of a co-culturing Arman-2 related organism, Mia14, with C. divulgatum PM4. Moreover, the authors also report on in situ possible interactions between these organisms, potential metabolic dependencies on hosts and the evolutionary trajectory from the Last DPANN ancestor to Mia14.

This is an exciting report regarding one of the "new" archaeal lineages from where little is known. However I have several concerns regarding its publication as is.

My first comment to this paper is that it should be revised by a native speaker in order to shorten sentences, correct grammar and make it easier to follow.

Secondly, I was unable to judge how the metabolic reconstruction of what is present or not in the genome was performed. Which pathways were searched for regarding e,g. cofactor biosynthesis, substrate-level phosphorylation? How was the presence/absence of a gene determined? I am sure that all the results are correct but more details in the methods/results section and supplementary material should be provided.

For instance, in the respiration section the authors talk about the absence of complex I to IV, including (and well) the search for alternative complex III (references missing). Did they also look for other enzymes belonging to anaerobic respiratory chains? In fact, in the genome provided by the authors there is a sulfide:quinone oxidoreductase (an FAD containing enzyme) that might (or not) work with the bd oxidase for the regeneration of the quinone/quinol pool. And regarding bd oxidase, the authors are surprised by its presence with no quinone biosynthesis pathways. I would go even further, I was also surprised because it is a heme dependent enzyme and heme biosynthesis and canonical heme import seems to be also absent from the genome. The same is true for the NAD-dependent D-glyceraldehyde dehydrogenase dependent enzyme the authors refer to.

I understand the appealing idea of the import of cofactors, energy and even DNA from Cuniculiplasma divulgatum through the type IV pili, surface proteins and membrane channels, but the presence of an ATP synthase (lines 398-399) per se does not necessarily imply the necessity of membranar complexes establishing a proton gradient, since the enzyme is reversible and can operate in both directions. If all the cofactors necessary for the proper operation of e.g. bd oxidase and NADH dependent enzymes would be acquired from C. divulgatum, why not all the ATP as well?

Many additional information regarding the methods are missing. I am sure that all the analysis was correctly done but it is hard to judge if for e.g. ProteinOrtho and IslandViewer3 the only information given, is a short sentence in the figure legends. In the case of IslandViewer3, how many Genome Islands were obtain? And by which method(s)?
Was query coverage ever taken into account in all taxonomic affiliations or just the high E-value of 10-6? Those are important details to access the validity of the results.

Minor: figure 4 and 5 are exchanged.

Reviewer #3 (Remarks to the Author):

This is an important manuscript that reports on an intriguing microbial group (so-called

Arman-like archaea), that has not yet been cultivated. Although partial genomes from metagenomes have been reported, since only metagenomic data so far has been available, complete inference of the metabolism and possible host dependencies of these archaea has been unavailable. The cultivation of this novel archaeal group reported in this manuscript now changes all that.

The authors report on the co-culturing of ARMAN-2-related organism, Mia14, with Cuniculiplasma divulgatum PM4 during the course of isolation. This co-culture allowed the assembly of a complete genome, from which metabolic completeness (or not) of specific pathways, and possible metabolic dependencies (for example in TCA, glycolysis, quinone biosynthesis, purine, pyrimidine, lysine, methionine, arginine, asparagine, alanine, aspartate, leucine, isoleucine, threonine, phenylalanine, tyrosine, and tryptophan biosynthesis) , could be inferred. Furthermore, analysis of several stains of the host along with shotgun metagenomic data indicated significant lateral gene transfer has likely occurred between the host(A) and the Arman-like archaeal parasite(s). The genomic assembly, analyses and metabolic inferences and conclusions appear generally sound, and provide important new information on the nature of Arman-like archaea, and their host-parasite interactions.

All in all this paper presents important new data on an intriguing group, as well as the power of the combination of methodology and synthesis using cultivation-dependent and cultivation independent techniques.

Reviewers' comments:

Reviewer #1 (Remarks to the Author):

Golyshina et al obtain a coculture of ARMAN belonging to Micrarchaeota phylum with that of member of the Thermoplasmates, using microscopy they show that they are likely in contact with one another as has been shown in nature. From this culture a complete genome of the ARMAN was obtained and used to look at gaps in metabolisms and gene transfer. Overall I found the paper to be fairly well written, a bit over wordy at times. The discussion about missing genes is relevant and interesting given the subject. However, many of the findings are similar to what has been seen in other DPANN, and they didn't not cite this work, Castelle et al. 2015 Current Biology. I think that this does advance our understanding of DPANN archaea, I just think the findings need to be placed in better context of what has been shown recently. There also needs to be a more robust sampling of genomes included in the phylogenetic analyses.

As suggested, we have commented on the paper of Castelle et al., 2015 in the introduction. In the Results (line 259) we have also emphasised on major differences in predicted metabolism of Mia14 as compared with representatives of "*Woesarchaeota*" and "*Diapherotrites"* even though the latter are phylogenomically very distant from Mia14. Please also see the comments below.

My other big issue is that few genomes (Woesearchaeota, Pacearchaeota and others from Castelle) were included in the analyses to look at gene transfer. If you are going to say that ARMAN has had more LGT that other DPANN I think the full set of available genomes should be included. This is really interest analyses, however.

Many genome comparisons require either completely sequenced genomes or unbiased samples thereof. In this work we limited our analyses to archaeal genomes that are complete or nearly complete according to our criteria (53 out of 56 universally conserved ribosomal proteins are readily identifiable among the annotated gene set). Unfortunately, just three "Woesearchaeota" but no "Pacearchaeota" representatives satisfied these criteria and were omitted from comparison. We believe that the consistency provided by our approach more than adequately compensates for the reduced coverage.

I find the title difficult to read and interrupt.

As suggested, we have changed the title for:
"ARMAN-related sentinel of *Cuniculiplasma* spp. in the laboratory culture and in the environment "

Why are Loki and Thor shown in bold in figure 4? These are not even discussed in the text.

> We have changed the figure as suggested (removed the boldface). Inclusion of these two candidate phyla was consistent with the quality of their genomic assemblies, along with other (in total, 285) archaea with complete and almost complete genomes where 53 of 56 ribosomal proteins could be identified. Majority of these genomes are situated in the collapsed branches and are not discussed either, as we believe this would dilute the paper with the information of a lower relevance.

The individual DPANN phyla should be labeled in this tree, fig 4. Especially which MIa44 belongs to! It is Micrarchaeota or Parvarchaeota?

> The formal taxonomic affiliation of Mia14, as well as the formal classification of "DPANN" organisms, "Micrarchaeota" and "Parvarchaeota" remains unresolved as of the time of this writing. To-date, none of these names are approved by the International Committee on Systematics of Prokaryotes, which is the only recognised body overseeing nomenclature of prokaryotes and setting up the rules by which microbial taxa are named. In relation to the Reviewer's specific question of affiliation of Mia14 to the candidate phyla proposed earlier, we have stated in the text (lines 158-178) that according to 16S rRNA gene sequence (s. the Manuscript), Mia14 shares about 75% sequence identity with cultured but fairly distant *Nitrososphaera* and *Methanosaeta* spp. (*Thaum*- and *Euryarchaeota*, respectively) and 92% with uncultured 'ARMAN-2' of *Ca*. "Micrarchaeota". The genome of the latter is however poorly resolved and could not be included into the downstream analysis. According to the ribosomal proteins sequences-based analysis shown on the Figure 4, we can comment that ("*Ca*. Iainarchaeum andersonii" from "*Ca*. Diapherotrites") seems the most related to Mia14, although their affiliation to the same phylum is not obvious (s. the Figure below; this figure is done for review purpose only in order to address the Reviewer's query about affiliation of Mia14).

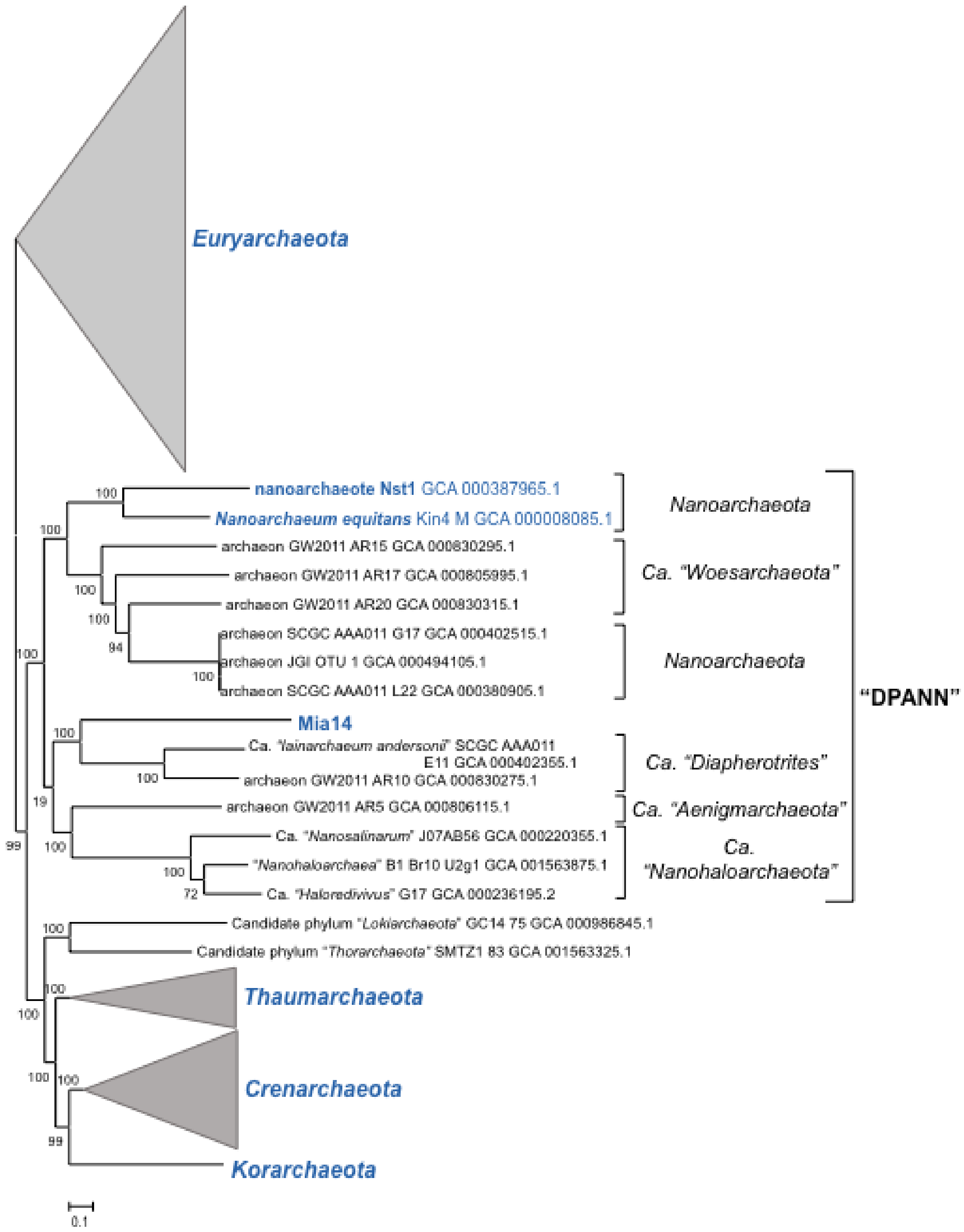

Furthermore, e.g. "Woesarchaeota" (AR17, AR15 and AR20 from Castelle et al., 2015) are clustered within the recognised phylum *Nanoarchaeota*, which casts some doubt in the validity of "Woesearchaeota" as a candidate phylum separate from *Nanoarchaeota*. We therefore intentionally avoided or reduced the use of these not recognised names of taxa of an unclear taxonomic ranking.

Furthermore, as this work is focused on a particular genome and is not intended to resolve the broader issues (including taxonomy), we believe that we provide enough phylogenomic context to make the position of Mia14 sufficiently clear without entering the debate on the formal issues.
In our opinion, we need to respect the recognised nomenclature and prevent multiplying controversy. Nevertheless, we leave this upon the Editor to decide if we shall stick with the tree format (Figure 4 re-submitted) or with the Figure version presented on the previous page in this document.

Also this tree lacks other ARMAN genomes, that is a must! This tree is actually missing lots of DPANN, but it needs to include other ARMAN genomes at least.

Please see the comment above. Only genomes that have passed our criteria for completeness were included. This is especially relevant to Figure 4 as the tree is constructed from the concatenated alignments of ribosomal proteins, so inclusion of highly incomplete genomes would be detrimental.

It's phylogenetic position in the DPANN phyla, which phylum, should clearly stated in the text as well.

Please see the comment above. We believe that i) as long as its relative position is known, the formal affiliation (as a member of a previously described phylum, or a candidate for a novel phylum) is of secondary importance and ii) this work is not the best place to attempt resolving this question.

Reviewer #2 (Remarks to the Author):

The paper by Golyshina et al report on the first ungapped genome of a co-culturing Arman-2 related organism, Mia14, with C. divulgatum PM4. Moreover, the authors also report on in situ possible interactions between these organisms, potential metabolic dependencies on hosts and the evolutionary trajectory from the Last DPANN ancestor to Mia14.

This is an exciting report regarding one of the "new" archaeal lineages from where little is known. However I have several concerns regarding its publication as is.

We thank the Reviewer 2 for this very positive assessment of our manuscript and for his/her constructive critique.

My first comment to this paper is that it should be revised by a native speaker in order to shorten sentences, correct grammar and make it easier to follow.

Done

Secondly, I was unable to judge how the metabolic reconstruction of what is present or not in the genome was performed. Which pathways were searched for regarding e,g. cofactor biosynthesis, substrate-level phosphorylation? How was the presence/absence of a gene determined? I am sure that all the results are correct but more details in the methods/results section and supplementary material should be provided.

We have added details into these sections.

For instance, in the respiration section the authors talk about the absence of complex I to IV, including (and well) the search for alternative complex III (references missing).

We have added missing references into this section.

In fact, in the genome provided by the authors there is a sulfide:quinone oxidoreductase (an FAD containing enzyme) that might (or not) work with the bd oxidase for the regeneration of the quinone/quinol pool.

Genomic context of ORF MIA14_0109 does not allow to assign the role of putative sulfide:quinone oxidoreductase to this protein. Considering the absence of quinone biosynthesis, it remains unclear if quinone cycle could exist in this organism at all.

Did they also look for other enzymes belonging to anaerobic respiratory chains? In fact, in the genome provided by the authors there is a sulfide:quinone oxidoreductase (an FAD containing enzyme) that might (or not) work with the bd oxidase for the regeneration of the quinone/quinol pool. And regarding bd oxidase, the authors are surprised by its presence with no quinone biosynthesis pathways. I would go even further, I was also surprised because it is a heme dependent enzyme and heme biosynthesis and canonical heme import seems to be also absent from the genome. The same is true for the NAD-dependent D-glyceraldehyde dehydrogenase dependent enzyme the authors refer to.

We have looked for molybdoenzymes and multiheme *c*-type cytochromes as putative determinants of sulfur, nitrogen and Fe(III) compounds respiration, but even we have found them, the organism is highly unlikely to possess an active anaerobic respiratory chain since proton-translocating complexes are absent in the genome.

Re. the import of hemes: generally, in bacteria such systems can be very diverse and were extensively studied for pathogenic bacteria that have at least six canonical variants of import of whole hemes (e.g. Choby JE, Skaar EP. Heme Synthesis and Acquisition in Bacterial Pathogens. J Mol Biol. 2016 Aug 28;428(17):3408-28. doi: 10.1016/j.jmb.2016.03.018.). However, the heme import pathways are very scarcely studied for archaea. In addition, we have assessed biosynthesis of porphyrins needed for biogenesis of hemes b and d, and could not find anything.

Considering all above, we have therefore modified the part on heme dependent enzyme and heme biosynthesis as follows (Line 421, Discussion):

"In this view, besides the possibility of completely novel heme biosynthesis pathway in this archaeon, the only way for proper assembly of the cytochrome *bd* complex is the incorporation of exogenous hemes. Accumulation of exogenous hemes in the membrane, which is capable of complementing the growth of heme-deficient organisms, has been demonstrated for pathogenic bacteria[40]. Considering that hemes *b* and *d* bind covalently to apoproteins and that the heme-binding amino acids are localised close to the surface of the cell membrane in cytochrome *bd* complexes[31], it seems possible for Mia14 to acquire exogenous hemes from *Cuniculiplasma* spp. to assemble its only PMF-generating complex. It should be noted that the complete set of genes for canonical or non-canonical heme biosynthesis pathways is also absent in *Cuniculiplasma* strains PM4 and S5, although these aerobically respiring organisms possess heme-containing enzymes of the electron transfer chain[16]. It seems therefore possible that *Cuniculiplasma*, and probably Mia14, possess yet unknown mechanisms of heme biosynthesis."

I understand the appealing idea of the import of cofactors, energy and even DNA from Cuniculiplasma divulgatum through the type IV pili, surface proteins and membrane channels, but the presence of an ATP synthase (lines 398-399) per se does not necessarily imply the necessity of membranar complexes establishing a proton gradient, since the enzyme is reversible and can operate in both directions. If all the cofactors necessary for the proper operation of e.g. bd oxidase and NADH dependent enzymes would be acquired from C. divulgatum, why not all the ATP as well?

It is a very interesting suggestion, which was discussed as a possible scenario in *Nanoarchaeum equitans* (Morris et al., 2013). In case of Mia14 it must then rely on a constant significant source of ATP probably coming from (or shared by) *Cuniculiplasma*. There could be an option of the proton export at expenses of ATP, but then Mia14 would need the sustainable source of ATP, however it has no substrate phosphorylation pathways. Whatever the case, at this stage we would prefer to avoid excessive speculations.

Many additional information regarding the methods are missing. I am sure that all the analysis was correctly done but it is hard to judge if for e.g. ProteinOrtho and IslandViewer3 the only information given, is a short sentence in the figure legends. In the case of IslandViewer3, how many Genome Islands were obtain? And by which method(s)?

We have amended the missing information according to this recommendation.

Was query coverage ever taken into account in all taxonomic affiliations or just the high E-value of 10-6? Those are important details to access the validity of the results.

The procedure was performed literally as described in the methods ("the top BLAST hit with an e-value threshold of 10^{-6}") without any additional filters or criteria. Given the considerable extent of domain rearrangement in the prokaryotic genomes (roughly, 3-4% of genes were involved in domain fusions with several times as many in fissions in the course of the history of Archaea), we decided that designing a proper domain-aware procedure would be an overkill for this particular study, whereas using simple coverage criteria will result in more errors than simply taking the top hit at face value.

Minor: figure 4 and 5 are exchanged.

Corrected

Reviewer #3 (Remarks to the Author):

This is an important manuscript that reports on an intriguing microbial group (so-called Arman-like archaea), that has not yet been cultivated. Although partial genomes from metagenomes have been reported, since only metagenomic data so far has been available, complete inference of the metabolism and possible host dependencies of these archaea has been unavailable. The cultivation of this novel archaeal group reported in this manuscript now changes all that.

The authors report on the co-culturing of ARMAN-2-related organism, Mia14, with Cuniculiplasma divulgatum PM4 during the course of isolation. This co-culture allowed the assembly of a complete genome, from which metabolic completeness (or not) of specific pathways, and possible metabolic dependencies (for example in TCA, glycolysis, quinone biosynthesis, purine, pyrimidine, lysine, methionine, arginine, asparagine, alanine, aspartate, leucine, isoleucine, threonine, phenylalanine, tyrosine, and tryptophan biosynthesis) , could be inferred. Furthermore, analysis of several stains of the host along with shotgun metagenomic data indicated significant lateral gene transfer has likely occurred between the host(A) and the Arman-like archaeal parasite(s). The genomic assembly, analyses and metabolic inferences and conclusions appear generally sound, and provide important new information on the nature of Arman-like archaea, and their host-parasite interactions.

All in all this paper presents important new data on an intriguing group, as well as the power of the combination of methodology and synthesis using cultivation-dependent and cultivation independent techniques.

We thank the Reviewer 3 for this very positive assessment of our manuscript.

REVIEWERS' COMMENTS:

Reviewer #1 (Remarks to the Author):

I still have issues with the manuscript, however don't think they should stop it from being published.

To me the title isn't very compelling to a broad audience for Nat Comm.

I still think that ARMAN-2 needs to be included in the tree Fig 4. Micrarchaeota ARMAN-2 is a near complete genome so the cutoffs don't seem reasonable. You state in the response that it is "poorly-resolved". This makes no sense to me, in NCBI it is less than <10 scaffolds, and in our hands it has been closed. This genome is the most important genome to compare Mia14 to. There are also several new MIcrarchaeota genomes in NCBI that I high quality. I would check those to see if they meet your cutoffs. As this tree is, there are no other ARMAN included. This will looked upon poorly if published this way.

I also don't agree with the reasoning for delineated where the proposed DPANN phyla are in this tree. It simply helps the reader understand the tree. The ICSP guidelines for naming are outdated and need to be updated. Which is why they are not followed much anymore.

Reviewer #2 (Remarks to the Author):

The authors have addressed all of the concerns I had with the previous submission.

REVIEWERS' COMMENTS:

Reviewer #1 (Remarks to the Author):

I still have issues with the manuscript, however don't think they should stop it from being published.

To me the title isn't very compelling to a broad audience for Nat Comm.

(A) The title has been changed to:

Mysterious “ARMAN” archaea depend on association with euryarchaeal host in culture and *in situ*.

I still think that ARMAN-2 needs to be included in the tree Fig 4. Micrarchaeota ARMAN-2 is a near complete genome so the cutoffs don't seem reasonable. You state in the response that it is "poorly-resolved". This makes no sense to me, in NCBI it is less than <10 scaffolds, and in our hands it has been closed. This genome is the most important genome to compare Mia14 to. There are also several new MIcrarchaeota genomes in NCBI that I high quality. I would check those to see if they meet your cutoffs. As this tree is, there are no other ARMAN included. This will looked upon poorly if published this way.

(A) we agree that in the GenBank there is a small number of scaffolds related to ARMAN-2. This, however, per se, does not reflect the completeness of its genome. In fact, the total number of ribosomal proteins in all scaffolds of ARMAN-2 is 46, whereas the analysis, which resulted in the Fig. 4, was done with 53-56 ribosomal proteins. This is also a clear message about the genome incompleteness of ARMAN-2. In turn, the data on gene gains/losses presented in the Fig. 7a on the carbon copy of the phylogenetic tree in the Fig. 4, would not be extremely credible with the data from such an incomplete genome.
Nevertheless, according to the recommendation to reflect on ARMAN-2 relatedness of Mia14, we have produced a new 16S rRNA gene-based phylogenetic tree **Supplementary Figure 1. SSU rRNA phylogeny of Mia14 and closely related organisms of “DPANN” superphylum.** Apart from ARMAN-1 and -2, additional “Micrarchaeota” sequences have been included as well. This figure supports the statements of relatedness of Mia14 and ARMAN-2, and we hope that it substantiates their phylogenetic relationship discussed in the text.

I also don't agree with the reasoning for delineated where the proposed DPANN phyla are in this tree. It simply helps the reader understand the tree. The ICSP guidelines for naming are outdated and need to be updated. Which is why they are not followed much anymore.

(A) As suggested, in the Fig.4 we have added names of candidate clades (or lineages, or taxa, since their status as phyla is not obvious). Taxa are named according to NCBI

taxonomy. Related to ICSP guidelines for naming, we agree that it definitely needs updating. Nonetheless, as it was recently stated by Gribaldo and Brochier-Armanet (2012) and supported by Garrity and Oren (2012) "the identification of high-rank taxa in prokaryotes is essential to our knowledge of microbial diversity and evolution, but this has to be done within an ordered, coherent, and widely agreed frame". "It will be the task of the ICSP to coordinate the effort toward the establishment of an ordered system for the higher taxa, if possible within the framework of the Bacteriological Code." We would rather stick with the latter position, with all due respect to the Reviewer's view.

Reviewer #2 (Remarks to the Author):

The authors have addressed all of the concerns I had with the previous submission.

We are grateful to the the Reviewer 2 for all comments and his constructive critique in the first round of the review process and we are happy that all responses are accepted.
